# Effect of stocking density and effective fiber on the ruminal bacterial communities in lactating Holstein cows

**DOI:** 10.7717/peerj.9079

**Published:** 2020-04-29

**Authors:** Brooke A. Clemmons, Mackenzie A. Campbell, Liesel G. Schneider, Richard J. Grant, Heather M. Dann, Peter D. Krawczel, Phillip R. Myer

**Affiliations:** 1 Animal Science, University of Tennessee, Knoxville, TN, USA; 2 William H. Miner Agricultural Research Institute, Chazy, NY, USA

**Keywords:** Stocking density, Effective fiber, Rumen, Bacteria, Cattle, Microbes

## Abstract

Overstocking can be a major issue in the dairy cattle industry, leading to negative changes in feeding and resting behavior. Additional stress imposed and alterations in feeding behavior may significantly impact the rumen microbiome. The rumen microbiome is responsible for the successful conversion of feed to usable energy for its host. Thus, understanding the effects of stocking density on the rumen microbiome is imperative for further elucidation of potentially negative consequences of overstocking in dairy cattle. This study implemented a Latin Square design accounting for four pens of cattle and four treatment periods so that all treatment combinations were assigned to every pen during one period of the study. Two treatment factors, including two levels of physically effective neutral detergent fiber, achieved with addition of chopped straw, and stocking density (100% vs. 142%) of freestalls and headlocks, were combined and tested within a factorial treatment design. Within each pen, three or four cannulated cows (*n* = 15 total) were sampled for rumen content on the final day of each treatment period. Each treatment was randomly assigned to a single pen for a 14-day period. The V1–V3 hypervariable regions of the 16S rRNA gene were targeted for bacterial analyses. Variables with approximately normally-distributed residuals and a Shapiro–Wilk statistic of ≥0.85 were analyzed using a mixed model analysis of variance with the GLIMMIX procedure with fixed effects of feed (straw vs. no straw), stocking density (100% vs. 142%), and the interaction of feed × stocking density, and random effects of pen, period, feed × stocking × pen × period. Pen was included as the experimental unit in a given period and the sampling unit as cow. Variables included Shannon’s Diversity Index, Faith’s phylogenetic diversity index, chao1, observed OTU, and Simpson’s evenness E as well as most individual taxa. Data were analyzed in SAS 9.4 utilizing the GLIMMIX procedure to perform mixed model analysis of variance. If data were not normally distributed, a ranked analysis was performed. No differences were observed in α-diversity metrics by fiber or stocking density (*P* > 0.05). Beta diversity was assessed using weighted and unweighted Unifrac distances in QIIME 1.9.1 and analyzed using ANOSIM. No differences were observed in weighted (*P* = 0.6660; *R* = −0.0121) nor unweighted (*P* = 0.9190; *R* = −0.0261) metrics and *R* values suggested similar bacterial communities among treatments. At the phylum level, Tenericutes differed among treatments with an interaction of stocking density by feed (*P* = 0.0066). At the genus level, several differences were observed by treatment, including *Atopobium* (*P* = 0.0129), unidentified members of order RF39 (*P* = 0.0139), and unidentified members of family Succinivibrionaceae (*P* = 0.0480). Although no diversity differences were observed, taxa differences may indicate that specific taxa are affected by the treatments, which may, in turn, affect animal production.

## Introduction

Stocking density can have significant impacts on dairy cattle feeding behavior, feed intake, as well as comfort of the animals, which ultimately may lead to health issues and reduced milk production. Competition at feed bunks as a result of overstocking can result in reduced feed intake, particularly for cows lower in the social hierarchy (Friend, Polan & McGilliard, 1977; Grant & Albright, 2001; Olofsson, 1999). Reduced feed intake in dairy cattle has been associated with decreased milk production, particularly in the presence of multiple health issues (Bareille et al., 2003). Reduced feed intake can also result in decreased reproductive efficiency, leading to potentially millions of dollars lost in revenue for producers (Bellows, Ott & Bellows, 2002; De Vries, 2006; Dekkers, Ten Hag & Weersink, 1998). Understanding the effects of increased stocking density, particularly in conjunction with various nutritional strategies, is necessary to determine potential interventions or alternative methods for addressing issues that arise as a result of overstocking.

In dairy feeding systems, effective fiber is used to reduce the negative effects of high-grain diets. Easily-fermentable feedstuffs can result in the rapid production of short-chain fatty acids and lactic acid, causing sharp decreases in rumen pH which may lead to ruminal acidosis (Enemark, 2008; Enemark, Jorgensen & Enemark, 2002). In order to alleviate or reduce the risk of ruminal acidosis, physically effective neutral detergent fiber (peNDF) is incorporated into the diet (Enemark, 2008). Addition of peNDF also assists in the slowing of feed passage rate through the rumen, allowing for improved digestion of feeds and nutrient absorption (Jung & Allen, 1995; Zebeli et al., 2012). Maintaining optimal rumen health and function is critical when determining management and nutritional programs for high milk production. Yet, little is known about how the rumen microbial population responds to these stressors.

The rumen microbiome, or the collection of microbial DNA present in the rumen, provides insight into the dynamics of the ruminal environment. The rumen microbiota provide vital nutritional resources to the animal by breaking down feedstuffs into usable energy, proteins, and vitamins that are then made available to the host (Hungate, 1966). Variation in the rumen microbiome has been associated with changes in the ruminal environment, such as differences in pH, nitrogen cycling, protein turnover, and metabolite production (Clemmons et al., 2017; Hungate, 1975, 1966; Mao, Huo & Zhu, 2016; Min et al., 2002; Petri et al., 2012; Ribeiro et al., 2017). It is estimated that approximately 70% of glucogenic precursors (Seymour, Campbell & Johnson, 2005) as well as many other vitamins and nutrients required by cattle are supplied by the rumen microbiota, highlighting the importance of their role in animal health and production.

Diet contributes to great variation in the rumen bacterial community composition. In predominantly forage-based diet, Firmicutes tends to be dominant at the phylum level, whereas Bacteroidetes tends to be more prevalent in a concentrate- or grain-based diet (Carberry et al., 2012; Petri et al., 2014; Tapio et al., 2017). At the family level, bacteria that possess primarily fibrolytic and cellulolytic functions, such as Lachnospiraceae, Ruminococcaceae, and Fibrobacteraceae, tend to be dominant in animals consuming a forage-based diet (Thoetkiattikul et al., 2013). However, bacteria that exhibit amylolytic functions, such as Prevotellaceae and Flavobacteriaceae, are more abundant in animals that consume more rapidly-fermentable diets (Thoetkiattikul et al., 2013). The addition of peNDF should alter the rumen bacterial community composition given the effects of diet on the rumen microbiome; however, the effects of peNDF addition to the diet on the rumen bacterial community composition are still not well-understood (Shaw et al., 2016).

Microbiomes have significant impacts on their host, beyond the environment of which they occupy. Variability in microbiome composition and structure has been associated with various production-relevant phenotypes in cattle, such as the rumen microbes in feed efficiency (Myer et al., 2015a) and the uterine and vaginal bacterial communities in reproductive efficiency (Laguardia-Nascimento et al., 2015). Additionally, alterations in the rumen microbiome resulting in dysbiosis were found to have significant impacts on overall animal health (McCann et al., 2016; Petri et al., 2013). For example, increased relative abundances of *Streptococcus bovis* are often associated with the occurrence of ruminal acidosis (Khafipour et al., 2009; Russell & Hino, 1985). Given the strong relationship among the rumen microbiome, production-relevant phenotypes, and animal health, understanding how certain stressors related to management and nutritional strategies affect these microbial relationships is imperative to improving production. Therefore, the objective of this study was to determine the effect of two different stocking densities with or without straw on the rumen bacterial communities in dairy cows, with the hypothesis that overstocking would alter rumen bacterial community composition but additional peNDF would ameliorate this stress.

## Methods

### Animal housing and management

Animal care and handling protocols were approved by the William H. Miner Agricultural Research Institute Animal Care and Use Committee, approval number 2014AUR11.

Twelve multiparous and four primiparous ruminally-cannulated cows were evenly distributed across and assigned to 1 of 4 pens and housed at the William H. Miner Agricultural Research Institute (Chazy, NY, USA). The animals were housed in a naturally ventilated, saw-dust bedded 4-row freestall barn from November 12, 2014 to January 7, 2015. Pens were balanced for parity (2.2 ± 1.1; mean ± standard deviation), days in milk (DIM; 190 ± 103), and milk production (45.8 ± 8.2 kg/d) prior to the start of the study. Due to an infection, one primiparous cow was removed from the trial. It is unknown whether the treatments contributed to or exacerbated this response. The ruminally-cannulated cows were used for subsequent rumen bacterial community analysis. Each pen contained 17 head-to-head freestalls with facility design as described (Krawczel et al., 2012a). Cows were milked 3 times daily (approximately 13:00 h, 21:00 h, and 05:00 h) in a double-12 parallel parlor (Xpressway Parallel Stall System; Bou-Matic, Madison, WI, USA).

### Experimental design and treatments

Pens were assigned randomly to treatments in a 4 × 4 Latin square with 14-d periods using a 2 × 2 factorial arrangement of treatments (Campbell et al., 2015). The first 7 d served as an adaptation period to the treatment with the additional 7 d serving as the treatment period as this has been previously shown to induced stress response due to overstocking (Krawczel et al., 2012b). Two stocking densities (100% or 142%) and two diets (straw; S and no straw; NS) resulted in four treatments combinations: (1) 100NS, (2) 100S, (3) 142NS and (4) 142S. Chopped straw was added as an additional source of peNDF (Table 1). Stocking density was achieved through denial of access to both headlocks and freestalls (100%, 17 freestalls and headlocks per pen; 142%, 12 freestalls and headlocks per pen) as previously described (Krawczel et al., 2012b). Dietary forage consisted of 39.7% corn silage and 6.9% haycrop silage vs. 39.7% corn silage, 2.3% haycrop silage, and 3.5% chopped straw (dry matter; DM basis) for NS and S, respectively. Substitution of haycrop silage with straw resulted in peNDF content of 23.9 and 25.9% and undigested 240-h NDF (uNDF240) content of 8.5% and 9.7% of DM for NS and S diets, respectively. Diets were similar in forage composition except that a portion of haycrop silage was replaced with 3.5% chopped wheat straw (DM basis) for the S diet, which resulted in differences in peNDF. Each diet was formulated for 46 kg milk/d using NDS Professional© based on the Cornell Net Carbohydrate and Protein System model (v. 6.1; RUM&N Sas, Reggio Emilia, Italy) and met metabolizable energy (ME) and metabolizable protein (MP) requirements. Diets were mixed and delivered once daily at approximately 06:00 h with a Keenan mixing truck (Richard Keenan & Co. Ltd., Warwickshire, UK) and pushed up approximately 6 times daily. Rumen content was collected via cannula every 4 h for the final 24 h of each treatment. Rumen samples were collected from the ventral sac approximately 30 cm below the cannula opening. Rumen content was strained through a single layer of cheesecloth and frozen at −80 °C. Samples were thawed at room temperature for approximately 2 h and equal aliquots were removed from each time point from each cow during each treatment period. Aliquots were then combined to get one total sample from each cow, and vortexed briefly to mix.

**Table 1:** Ingredient composition and analyzed chemical composition (dry matter basis) of TMR samples for no straw (NS) and straw (S) diets.

Item	NS	S
Ingredient, % of DM		
Conventional corn silage	39.7	39.7
Haycrop silage	6.9	2.3
Wheat straw, chopped^1^	–	3.5
Citrus pulp, dry	4.8	4.8
Whole cottonseed, fuzzy	3.5	3.5
Soybean meal, 47.5% solvent	–	1.1
Molasses	3.2	3.2
Concentrate mix^2^	41.9	41.9
Chemical analysis, % DM		
DM, %	45.9 ± 0.4^3^	47.5 ± 0.5
CP	15.0 ± 0.3	15.1 ± 0.3
Soluble protein, % of CP	32.0 ± 0.8	28.2 ± 1.4
NDICP^4^	1.1 ± 0.0	1.1 ± 0.0
ADF	20.0 ± 0.3	20.1 ± 0.3
NDF	28.9 ± 0.5	31.7 ± 0.7
ADL	3.8 ± 0.1	3.8 ± 0.1
NFC	43.1 ± 0.4	43.7 ± 0.6
Starch	25.0 ± 0.4	25.3 ± 0.6
Starch digestibility (7-h), % of starch	73.3 ± 1.0	74.3 ± 0.5
Sugar	7.4 ± 0.3	8.1 ± 0.4
Fat	5.9 ± 0.2	5.7 ± 0.2
Ash	6.4 ± 0.2	6.4 ± 0.4
Ca	0.71 ± 0.20	0.72 ± 0.03
P	0.38 ± 0.00	0.38 ± 0.01
Mg	0.41 ± 0.00	0.40 ± 0.00
K	1.22 ± 0.03	1.16 ± 0.02
S	0.26 ± 0.01	0.26 ± 0.01
Na	0.45 ± 0.01	0.44 ± 0.01
Cl ion	0.50 ± 0.02	0.47 ± 0.01
Fe, mg/kg of DM	209 ± 9	212 ± 11
Mn, mg/kg of DM	86 ± 1	83 ± 2
Zn, mg/kg of DM	96 ± 1	94 ± 1
Cu, mg/kg of DM	19 ± 0	18 ±1
Net energy of lactation, Mcal/kg of DM	1.76 ± 0.01	1.75 ± 0.02
Physically effective NDF _>1.18 mm_, % of DM^5^	23.9	25.9
30-h uNDFom, % of DM^6^	13.1	14.9
120-h uNDFom, % of DM	9.0	10.2
240-h uNDFom, % of DM	8.5	9.7

**Notes:**

1 Hay-busted; hammer-mill chopping technique; mo. #H1100, Duratech Industries Inc., Jamestown, North Dakota.

2 Concentrate mix was composed of the following (% of DM): corn meal, finely ground (32.31), soybean meal 47.5 solvent (15.90), AminoMax (Afgritech LLC, Watertown, NY, USA; 14.28), flaked corn (12.72), Berga Fat F100 (Berg + Schmidt America LLC, Libertyville, IL, USA; 5.65), wheat red dog (4.77), canola meal solvent (3.98), Amino Enhancer (Poulin Grain Inc., Swanton, VT, USA; 3.88), calcium carbonate (2.39), sodium sesquicarbonate (1.62), salt (0.78), magnesium oxide (0.55), Meta Smart (Adisseo, Alpharetta, GA, USA; 0.35), trace mineral mix (contained Diamune SE concentrate (Diamond V; Cedar Rapids, IA, USA)); 58.33%, zinc sulfate, 14.04%, manganese sulfate, 13.64%, calcium carbonate, 5.50%, 30% ferrous sulfate, 5.40%, 58% Intellibond copper (Micronutrients, Indianapolis, IN, USA; 1.17%, mineral oil, 1.00%, 3% selenium, 0.53%, cobalt sulfate, 0.29%, and calcium iodate, 0.11%; 0.20), Urea (0.19), Select GH (Alltech, Inc., Nicholasville, KY, USA; 0.13), Gen 2-AjiPro-L (Ajinomoto Heartland, Inc., Chicago, IL, USA; 0.10), vitamins A, D and E premix (contained calcium carbonate, 78.77%, vitamin E, 18.00%, vitamin A 1000 kIU and vitamin D 200 kIU, 2.34%, mineral oil, 0.50%, Vitamin D, 0.14%; 0.06), Smartamine M (Adisseo, Alpharetta, GA, USA; 0.06), Zinpro Availa 4 (Zinpro Corporation, Eden Prairie, MN, USA; 0.05), vitamin E premix (contained 88.18 kIU vitamin E, 7.08 mg/kg Cu; 0.02), Probios Precise Concentrate (Chr-Hansen, Milwaukee, WI, USA; 0.02), and Rumensin 90 (Elanco Animal Health, Greenfield, IN, USA; 0.01).

3 Mean ± standard error.

4 Neutral detergent insoluble CP.

5 peNDF determined through methods described by Mertens (2002).

6 uNDFom determined through methods described by Tilley & Terry (1963) with Goering & Van Soest (1970) buffer modifications.

### DNA extraction and sequencing

Approximately 0.2 g of sample was then used to extract bacterial DNA. Genomic DNA was extracted using a modified method described by Yu & Morrison (2004). Samples underwent both mechanical and chemical lysis using ZR BashingBead Lysis Tubes (Zymo Research Corp., Santa Ana, CA, USA) using the TissueLyser II system (Qiagen, Hilden, Germany) for 3 min at 21 Hz with 4% (w/v) sodium dodecyl sulfate (SDS), 500 mM NaCl, and 50 mM EDTA, respectively. Following cell lysis, impurities were removed using 10 M ammonium acetate and nucleic acids precipitated using isopropanol. Following nucleic acid precipitation, RNA and proteins were removed using RNAse and proteinase K. Subsequently, QIAmp columns were used to remove undesired products and purify DNA from the Qiagen DNA Stool Mini Kit (Qiagen, Hilden, Germany). Genomic DNA was quantified using Nanodrop 1000 spectrophotometer (ThermoScientific, Wilmington, DE, USA).

The V1–V3 region of the 16S rRNA hypervariable gene was targeted for amplification for bacterial analysis using modified universal primers 27F (5′-Adapter/Index/AGAGTTTGATCCTGGCTCAG) and 519R (5′ Adapter/Index/GTATTACCGCGGCTGCTG) including TruSeq adapter sequences and indices. AccuPrime Taq high fidelity DNA Polymerase (Life Technologies, Carlsbad, CA, USA) was used as the DNA polymerase. Bacterial DNA was amplified using polymerase chain reaction (PCR) with the following conditions: initial denaturation period of 94 °C for 5 min followed by 22 cycles of a denaturation period of 94 °C at 30 s, annealing period at 58 °C for 1 min, and elongation period at 72 °C for 1 min 30 s, with a final elongation period of 72 °C for 10 min. Products were purified magnetically using AmPure bead purification (Agencourt, Beverly, MA, USA), confirmed via gel electrophoresis and bioanalyzer (Agilent Technologies, Santa Clara, CA, USA). Amplicon libraries were sequenced using the 2 × 300, v3 600-cycle kit on the Illumina MiSeq sequencing platform as prepared per manufacturer protocol (Illumina, Inc., San Diego, CA, USA) at the University of Tennessee, Knoxville Genomics Core.

### Sequence reads and processing

Sequence data is available from the NCBI Sequence Read Archive accession PRJNA527780. Sequence reads were first trimmed by removing sequences shorter than 300 bp as well as removing adapters/indices, and quality filtered at ≥Q25 using the Galaxy server (Afgan et al., 2018). Further processing was performed in Quantitative Insights Into Microbial Ecology (QIIME, version 1.9.1) (Caporaso et al., 2010). Chimeric sequences were identified and filtered using usearch61 (Edgar, 2010). Operational taxonomic units (OTU) that were identified as Cyanobacteria were removed as these most likely represent chloroplast and mitochondrial DNA (Gray, 1993; Gray, Burger & Lang, 1999), as well as singletons. Each sample was randomly subsampled to a depth of 25,000 sequences to avoid sequencing depth bias. Operational taxonomic unit identification was performed at 97% similarity using UCLUST module in QIIME, and taxonomy assigned using the Greengenes v13_8 16S rRNA database as a reference (Caporaso et al., 2010; DeSantis et al., 2006; Edgar, 2010; McDonald et al., 2012). Phylogenetic trees were built using FastTree (Price, Dehal & Arkin, 2009) for analysis of alpha- and beta-diversity. Alpha-diversity was assessed using Faith’s phylogenetic diversity index, chao1, observed OTU, Shannon’s diversity index, and Simpson’s Evenness E, with Good’s coverage determined to ensure adequate coverage. Good’s coverage was assessed by treatment group to ensure satisfactory coverage (≥0.98). Beta-diversity was determined using weighted and unweighted Unifrac distances (Lozupone et al., 2011) and used to generate principal coordinate analyses (PCoA).

### Statistical analyses

Alpha-diversity metrics as well as genus- and phylum-level relative abundances were assessed for normality using the UNIVARIATE procedure in SAS 9.4 (SAS Institute, Cary, NC, USA). Alpha diversity metrics were determined to be approximately normal based on a Shapiro–Wilk value of ≥0.85 as well as visual distribution of data in histogram and quintiles plot of residuals. Alpha diversity was measured using Faith’s phylogenetic diversity index, chao1, observed OTU, Shannon’s diversity index, and Simpson’s evenness E. Alpha diversity metrics were analyzed using a mixed model analysis of variance (ANOVA) with fixed effects of feed (straw vs. no straw), stocking density (100% vs. 142%), and the interaction of feed×stocking density, and random effects of pen, period, feed × stocking × pen × period. Pen was included as the experimental unit in a given period and the sampling unit as cow. The statistical model was analyzed using the GLIMMIX procedure of SAS 9.4. Genus- and phylum-level relative abundances were analyzed for normality in SAS 9.4. For values that were normally distributed, taxa were analyzed using the same model as those used for alpha-diversity metrics. Those with a non-normal distribution were first ranked and then analyzed using the same, previously described model. This method of analysis allows random effects to be properly accounted for while performing a ranked analysis, similar to Kruskal–Wallis test. Beta diversity metrics were analyzed in QIIME (Caporaso et al., 2010) using weighted and unweighted Unifrac distance matrices as well as ANOSIM with 999 permutations. Beta-diversity was visualized using PCoA and analyzed using ANOSIM using an OTU-centric approach to compare phylogenetic variation in the OTU among treatment groups. For all statistical analyses significance was determined using at *P* ≤ 0.05.

## Results

### Sequencing data

Sixty samples were sequenced, comprising 15 cows from each of the four periods. Bacterial DNA sequencing resulted in a total of 31,291,696 sequences, with 28,492,337 sequences present following quality control and chimera removal. With regard to treatment, the NS100 treatment resulted in a total of 7,891,481 sequences, 4,424,972 total sequences for S100, 4,652,929 total sequences for NS142, and 10,655,805 total sequences for S142 with averages and standard errors of the mean of 526,098.73 ± 25,689.00, 294,998.1 ± 45,148.39, 310,195.3 ± 42,931.73, and 710,387 ± 77,008.81 per treatment sample, respectively.

### Alpha- and beta-diversity

A total of 158,618 OTU were identified following binning at 97% similarity. Alpha-diversity was measured using Faith’s Phylogenetic Diversity, chao1, observed OTU, Shannon’s diversity index, and Simpson’s evenness E, with Good’s coverage used to measure and ensure adequate coverage. Alpha-diversity metric averages with SEM are presented in Table 2. No significant interactions (*P* > 0.05) were identified among any of the α-diversity metrics, nor were there any diet or stocking density main effects associated (Table 2).

**Table 2:** Alpha diversity metrics by treatment group.

Alpha diversity metric^a^	NS100^b^	NS142^b^	S100^b^	S142^b^
Faith’s phylogenetic diversity	62.21 ± 0.65	61.92 ± 0.82	60.57 ± 0.71	62.13 ± 0.70
Chao1	2,977.18 ± 38.23	2,969.98 ± 49.60	2,949.20 ± 61.88	2,962.75 ± 52.76
Observed OTU	2,642.80 ± 50.81	2,634.87 ± 55.11	2,639.00 ± 70.64	2,657.87 ± 59.93
Good’s coverage	0.99 ± 0.00	0.99 ± 0.00	0.99 ± 0.00	0.99 ± 0.00
Shannon’s diversity index	9.20 ± 0.03	9.20 ± 0.06	9.13 ± 0.08	9.17 ± 0.04
Simpson’s evenness E	0.07 ± 0.00	0.06 ± 0.00	0.06 ± 0.00	0.06 ± 0.00

**Notes:**

a No statistical significance (*P* > 0.05).

b Mean ± SEM, NS, no straw; S, Straw; 100, 100% stocking rate; 142, 142% stocking rate.

**Figure 1:**
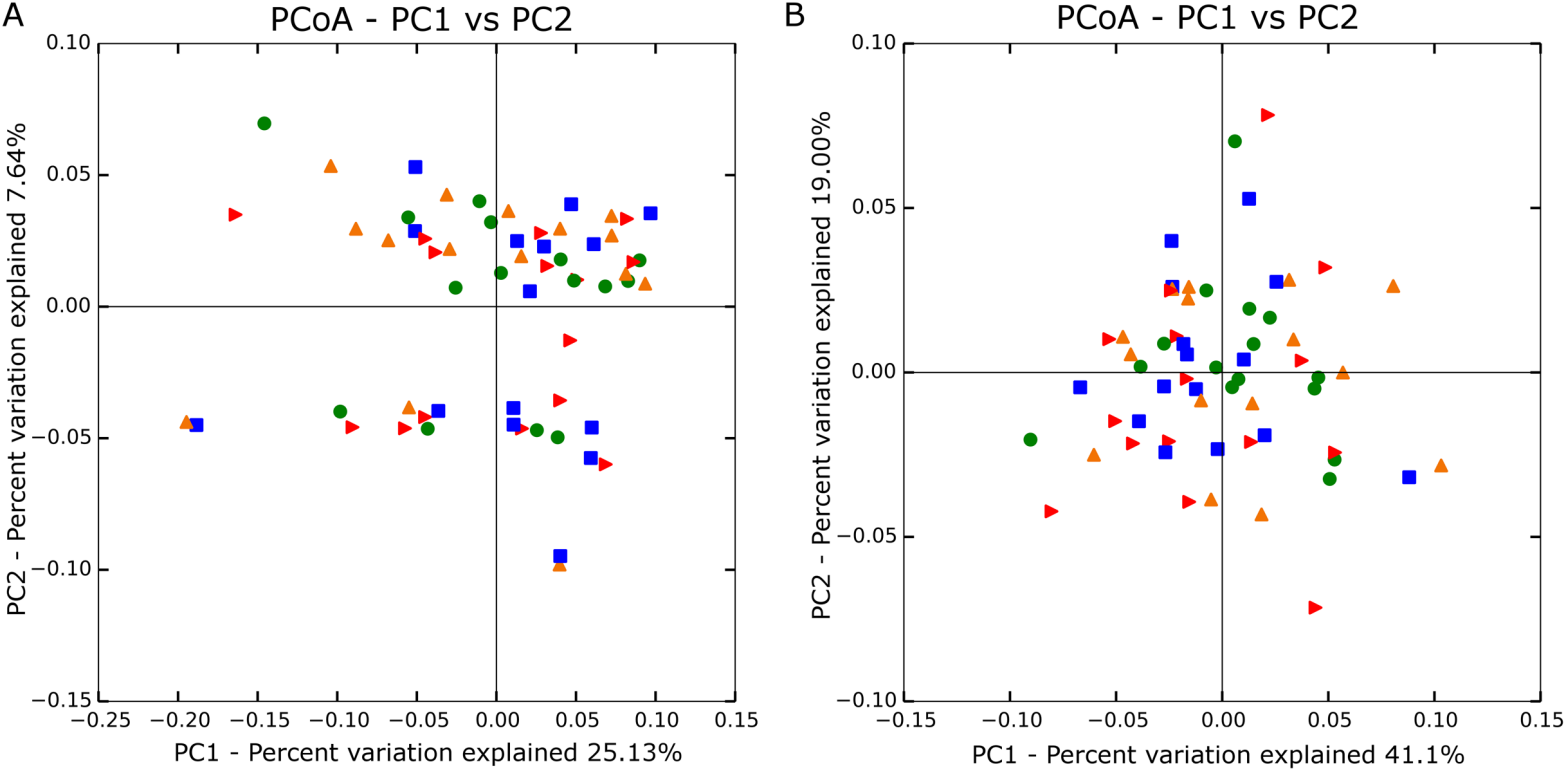
Principle coordinate analysis based on unweighted (A; *n* = 60) and weighted Unifrac distances (B; *n* = 60). Blue square is no straw at 142% stocking density, red arrow is no straw at 100% stocking density, green circle is straw at 142% stocking rate, and orange triangle is straw at 100% stocking rate.

Principal coordinate analyses using both unweighted (Fig. 1A) and weighted (Fig. 1B) Unifrac distances showed no significant grouping by treatment. Analysis of similarity also confirmed no differences in β-diversity among treatment groups based on unweighted (*R* = −0.026; *P* = 0.919) and weighted values (*R* = −0.012; *P* = 0.666).

### Taxonomic analyses

**Figure 2:**
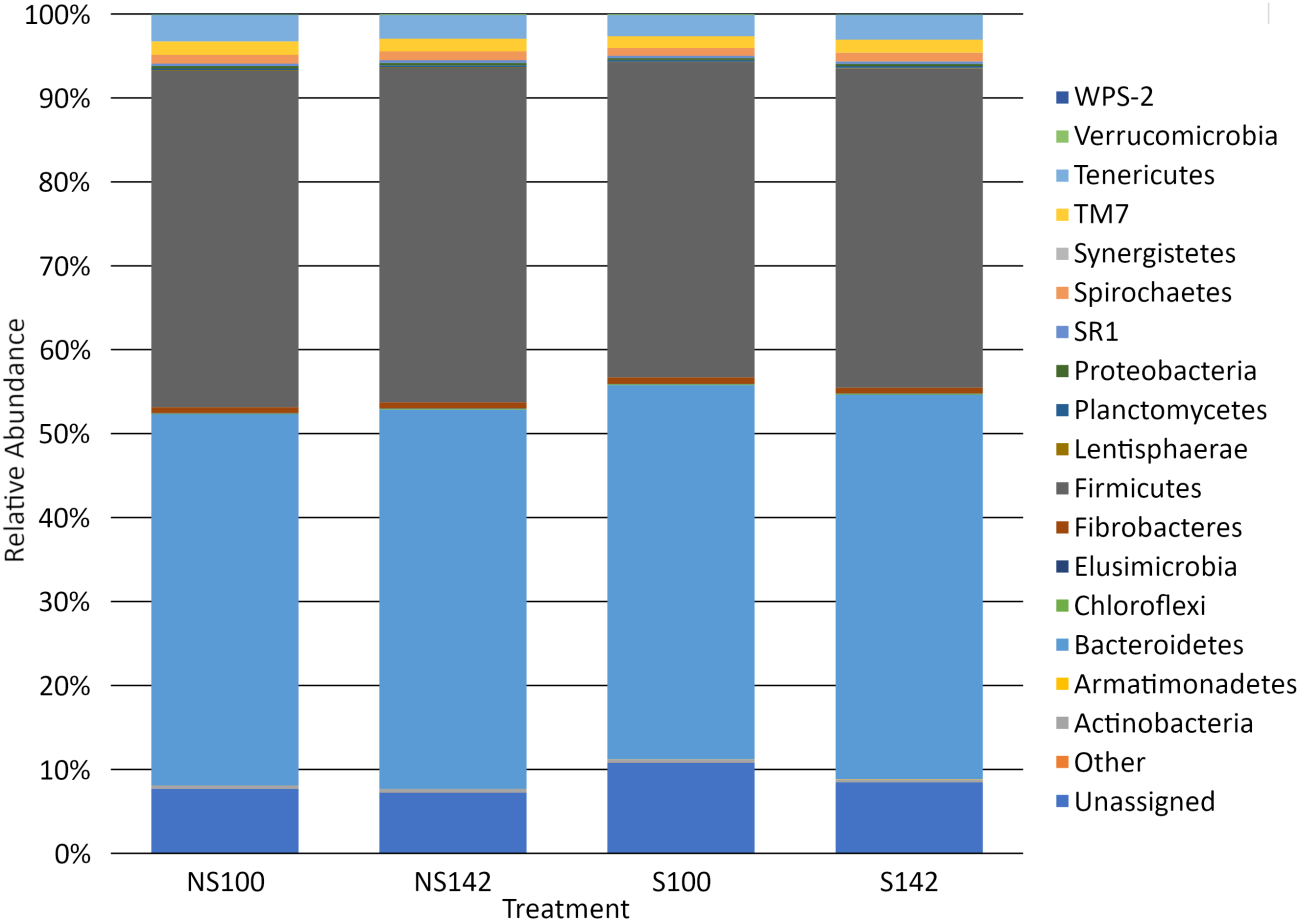
Bar chart of relative abundance of phyla by treatment. S142, straw at 142% stocking density; NS142, no straw at 142% stocking density; S100, straw at 100% stocking density; NS100, no straw at 100% stocking density.

Among all treatment combinations, Bacteroidetes was the most dominant phylum, with an average relative abundance of 44.94 ± 1.42%, followed by Firmicutes (38.89 ± 1.23%), unassigned taxa (8.55 ± 1.17%), Tenericutes (2.85 ± 0.12%), TM7 (1.52 ± 0.09%), and Spirochaetes (1.01 ± 0.06%), with all other phyla representing less than 1% relative abundance. Tenericutes was the sole phylum that differed among treatment groups, with an interaction of stocking density by feed (*P* = 0.01). When stocking density was 100%, the relative abundance was 3.13 ± 0.15% in the absence of straw and 2.53 ± 0.12% in the presence of straw (Table 3). When stocking density was 142%, the relative abundance of Tenericutes was 2.80 ± 0.09% in the absence of straw and 2.93 ± 0.11% in the presence of straw (Table 3). Fisher’s LSD groupings are presented in Table 3. A bar chart representing the relative abundance of phyla by treatment combination is presented in Fig. 2.

**Table 3:** Significant taxa differences by treatment combination, diet, and stocking density.

Taxa level	Taxon	Treatment group				*P* values		
		NS100*	NS142*	S100*	S142*	Interaction	Diet	Stocking
Phylum	Tenericutes	3.13 ± 0.15^A^	2.80 ± 0.09^AB^	2.53 ± 0.12^B^	2.93 ± 0.11^A^	0.01	0.07	0.82
Phylum	Unassigned	7.70 ± 1.14^B^	7.66 ± 1.21^B^	10.80 ± 1.36^A^	7.94 ± 1.02^B^	0.07	0.04	0.11
Order	RF39	2.26 ± 1.39 × 10^−1,A^	1.94 ± 6.14 × 10^−2,AB^	1.82 ± 9.64 × 10^−2,B^	2.21 ± 1.10 × 10^−1,AB^	0.02	0.50	0.88
Family	Succinivibrionaceae^†^	1.79 × 10^−2^ ± 5.82 × 10^−3,A^	1.65 × 10^−2^ ± 6.12 × 20^−3,A^	8.75 × 10^−3^ ± 1.59 × 10^−3,B^	5.52 × 10^−3^ ± 7.99 × 10^−4,B^	0.66	0.05	0.22
Family	Anaeroplasmataceae	4.46 × 10^−2^ ± 9.92^−3,A^	4.80 × 10^−2^ ± 1.28 × 10^−2,A^	2.28 × 10^−2^ ± 6.41 × 10^−3,B^	2.58 × 10^−2^ ± 7.75 × 10^−3,B^	0.82	0.02	0.81
Family	Caulobacteraceae	1.79 × 10^−2^ ± 1.77 × 10^−3,A^	1.80 × 10^−2^ ± 2.31 × 10^−3,A^	1.34 × 10^−2^ ± 2.09 × 10^−3,B^	1.27 × 10^−2^ ± 1.68 × 10^−3,B^	0.94	0.03	0.85
Genus	Atopobium	1.58 × 10^−3^ ± 6.12 × 10^−4,B^	4.89 × 10^3^ ± 9.34 × 10^−4,A^	2.14 × 10^−3^ ± 5.07 × 10^−4,B^	2.89 × 10^−3^ ± 8.54 × 10^−4,A^	0.19	0.47	0.04

**Notes:**

* Mean ± SEM, NS, no straw; S, straw; 100, 100% stocking rate; 142, 142% stocking rate.

† *P* values based on ranked data.

^AB^Within-row differences indicate statistically different groups.

**Figure 3:**
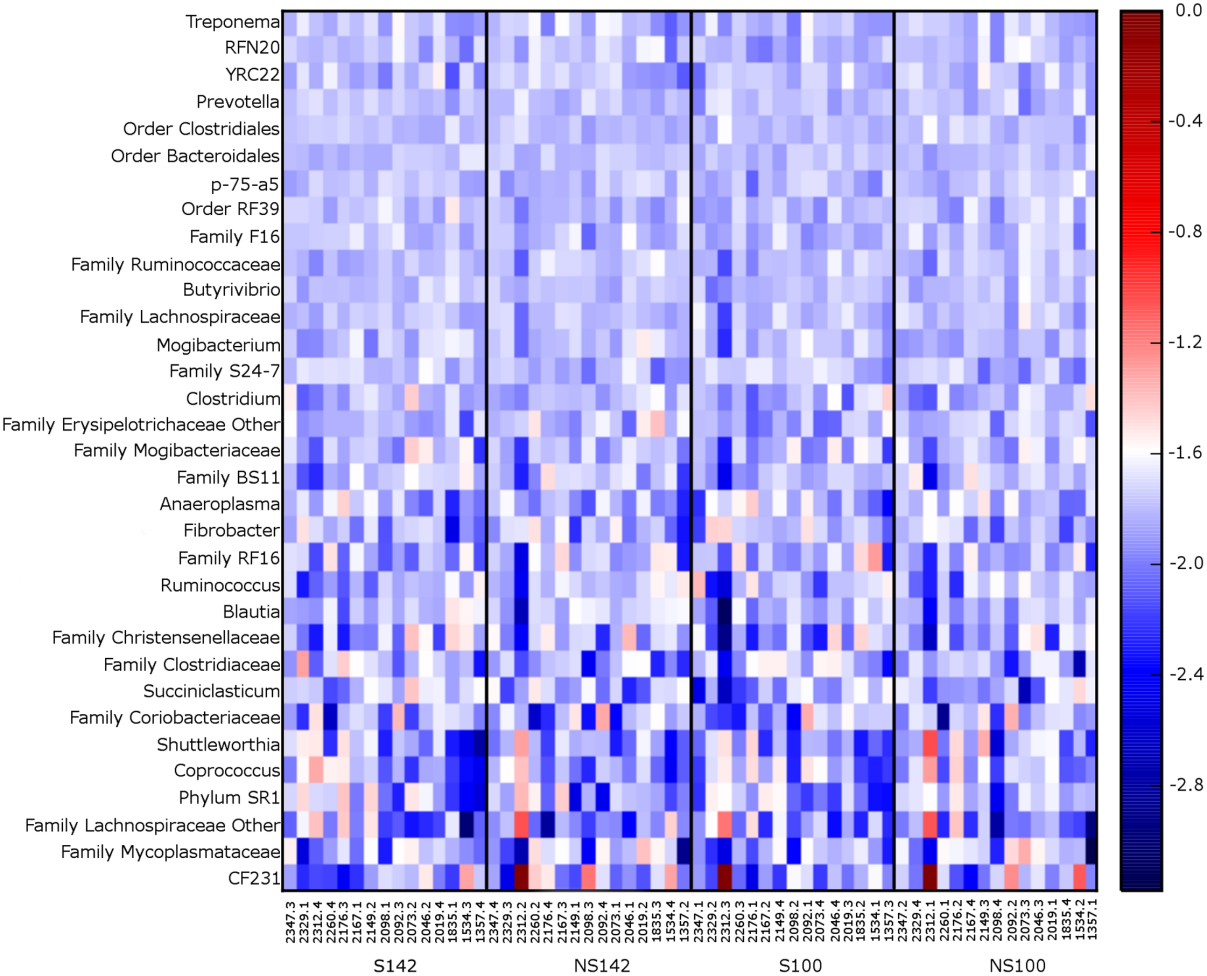
Heatmap of 0.1% most relative abundant genera for each cow. Organized by treatment (from left to right—straw at 142% stocking density, no straw at 142% stocking density, straw at 100% stocking density, and no straw at 100% stocking density).

At the genus level, the genera representing ≥1% relative abundance across all treatment combinations included, *Prevotella* (25.67 ± 1.32%), unclassified members of the order Clostridiales (10.00 ± 0.39%), unclassified members of the family Ruminococcaceae (8.60 ± 0.46%), unclassified members of the order Bacteroidales (7.30 ± 0.28%), *Ruminococcus* (6.92 ± 0.75%), unclassified members of the family S24-7 (6.55 ± 0.39%), unclassified members of family Lachnospiraceae (3.64 ± 0.15%), *Butyrivibrio* (2.55 ± 0.12%), unclassified members of order RF39 (2.06 ± 0.10%), unclassified members of family F16 (1.52 ± 0.08%), unclassified members of the family RF16 (1.51 ± 0.18%), other members of the family Lachnospiraceae (1.26 ± 0.27%), *RFN20* (1.24 ± 0.09%), and *YRC22* (1.02 ± 0.05%). A heatmap of genera illustrating relative abundances by treatment is available in Fig. 3. Several taxa differed among treatment combination groups. Average relative abundances with SEM of significantly different phyla and genera are available in Table 3.

## Discussion

This study measured the short-term effects of two different levels of stocking density with or without the presence of additional straw to represent additional peNDF on the rumen bacterial community structure and composition. Overstocking can be an issue in the dairy cattle industry, leading to reduced performance and decreased animal health (Bareille et al., 2003; Friend, Polan & McGilliard, 1977; Grant & Albright, 2001; Olofsson, 1999). Furthermore, the high grain diet typically fed to dairy cattle can result in negative physiological responses caused by rapid decreases in pH. Additional peNDF is often used in the dairy industry to counteract the negative effects of high grain diets that are common in the dairy industry by preventing rapid decreases in rumen pH from rapid fermentation of feeds (Enemark, 2008). Given the significant role of the rumen microbiome in feed utilization, this study determined the effects of the stocking density on the rumen at a microbial community level through diversity as well as at taxonomic levels.

In this study, no differences were observed in either α- or β-diversity of the bacterial communities among treatments. This was unexpected given that different feedstuffs and their composition, particularly types and levels of forage and starch, have previously had a significant impact on rumen microbial populations. A study conducted by Thoetkiattikul et al. (2013) examined the effect of varying levels of dietary fiber and starch on the rumen bacterial populations of dairy cows (Thoetkiattikul et al., 2013). The authors found that greater concentrations of either fiber (88% fiber and 2% starch) or starch (57.5% fiber and 21% starch) resulted in decreased α-diversity metrics, including those metrics for both richness and diversity, compared with the median diet (76% fiber and 10% starch) (Thoetkiattikul et al., 2013). This trend has also been observed in other studies, including those conducted in beef cattle. In a study conducted by Fernando et al. (2010), animals fed a high-grain (80% grain/20% forage) diet had lower α-diversity as measured by chao1 and ACE than those on high-forage diets (80% forage/20% grain) (Fernando et al., 2010). However, these previous studies used much more extreme diets regarding high starch, low fiber vs. low starch, high fiber, and likely contributed to the different responses. Because no differences in α- or β-diversity were observed in the current study, these metrics may not be as important for understanding the impacts of stocking density or additional peNDF on the rumen microbiome. However, lack of diversity could be attributed to time of last meal or little difference in peNDF. Although the animals were sampled every 4 h for 24 h, and the samples pooled by day per animal, this greater variation by day per sample may impact the ability to detect diversity differences. Further, if animals were exposed to treatment for longer durations of time or a greater number of animals were used, differences may be observed.

Alternatively, the effect of stocking density was less frequently studied. One of the few studies that included the effect of any stocking variable, such as stocking intensity, on rumen bacterial communities was conducted by McCann et al. (2014). The authors analyzed the effects of different models of residual feed intake (RFI) and stocking intensity on the rumen bacterial populations in beef bulls (McCann et al., 2014). The results of the beef bull study concur with the results of the current study, suggesting that stocking variables may not significantly impact the rumen bacterial diversity (McCann et al., 2014). However, as McCann et al. (2014) mentioned, individual animals possess their own unique rumen microbiomes, and the number of animals used in the study may not have been enough to overcome the amount of individual animal variation.

Although no differences were observed among treatment groups in bacterial community diversity, both phylum- and genus-level divergences were measured among treatment groups by interactions. At the phylum level, Tenericutes was the sole taxon that differed among treatment groups. Tenericutes are frequently found as a component of the rumen microbiome in cattle (Creevey et al., 2014; Jami & Mizrahi, 2012; Myer et al., 2015b), particularly in adult cattle (Jami et al., 2013). Pitta et al. (2014) observed that relative abundances of Tenericutes changed as a result of diet changes through the transition period in dairy cattle (Pitta et al., 2014). Tenericutes are small, prokaryotic cells that do not possess cell walls, allowing them to exhibit flexibility in their morphology (Brown, 2010). The only class found in Tenericutes is Mollicutes, of which some of the more notable members are *Mycoplasma* (Ludwig, Euzéby & Whitman, 2010). The differences observed in Tenericutes could possibly be the result of normal temporal fluctuations that occur in the rumen bacterial communities (Clemmons et al., 2019; Pitta et al., 2014). Additionally, one of the members of Tenericutes, Anaeroplasmataceae, also varied as a result of treatment group. Further studies assessing stocking density may reveal more information, especially given that few studies exist investigating the relationship between stocking density and the rumen microbiome in cattle.

At the genus level, several taxa differed by treatment group. *Atopobium*, a member of Actinobacteria, is commonly found in the rumen (Mao et al., 2013; Petri, 2013; Šuľák et al., 2012). Although the specific role of *Atopobium* in the rumen is not well known (Šuľák et al., 2012), it does appear to be associated with diet variation, particularly with greater-concentrate diets and instances of ruminal acidosis (Mao et al., 2013; Petri, 2013). Abundance of *Atopobium* in the present study does not appear to be associated with presence or absence of sufficient peNDF, but may be a result of overstocking, based on the average relative abundances by treatment. Similarly to ruminal acidosis, overstocking may represent a source of stress on the animal (Krawczel et al., 2012a; Webster, 1983). Indeed, greater stocking rates may provide a source of subclinical stress and, when combined with an additional stressor such as low fiber content in the diet, could result in altered rumen microbiomes (Campbell et al., 2015). Relative abundances of *Atopobium* may increase in response to physiological or environmental stress (Mao et al., 2013); however, more research needs to be conducted to confirm this theory.

Unclassified members of the order RF39 also varied by treatment. Members of order RF39 appear to be present in the rumen (Jami & Mizrahi, 2012) as well as the gut microbiomes of other species (Goodrich et al., 2014; Lin et al., 2013). In a study conducted by Jami & Mizrahi (2012), the authors attempted to interrogate the relationships among the rumen microbiome, feed efficiency, and milk production in dairy cattle (Jami & Mizrahi, 2012). The authors found a tendency for positive correlation between an unclassified genus of RF39 and RFI, a measure of feed efficiency, suggesting that even at lower abundances this taxon may contribute to host physiological variation (Jami, White & Mizrahi, 2014). Additional studies support the positive relationship between RF39 and RFI in dairy cattle (Jewell et al., 2015). Greater RFI is considered less efficient because animals are consuming more feed than would be expected given their metabolic body weight and average daily gain (Koch et al., 1963). Although stocking density is intuitively different than feed efficiency, the presence and abundance of RF39 in dairy cattle may suggest a less efficient or even maladaptive rumen microbiome. Although this specific trend was not necessarily observed in this study, greater number of animals and longer exposure to overstocking may reveal the relationship among RF39 and stocking density.

The third genus-level taxon that differed by treatment was unclassified members of family Succinivibrionaceae. Members of Succinivibrionaceae are Gram-negative, succinate-producing bacteria commonly found in the rumen of livestock ruminants (Stackebrandt & Hespell, 2006). Bacteria from Succinivibrionaceae are most often found in greater abundance in the rumen of animals fed diets with greater amounts of starch, or more readily fermentable feed (Bryant et al., 1958; Bryant & Small, 1956; Wozny et al., 1977). These results support the observations in the current study, in which relative abundances of unclassified members of the family Succinivibrionaceae were greater in the diets that did not contain the additional straw and greater peNDF content. Additionally, in the current study, relative abundances of Succinivibrionaceae numerically decreased with greater stocking density in both diet treatments. A study conducted by McCabe et al. (2015) analyzed the effects of feed restriction on bulls fed high-grain diets. The authors found that members of Succinivibrionaceae decreased following feed restriction (McCabe et al., 2015). Although increased stocking density does not necessarily result in decreased feed intake for all animals, it could result in an altered feed intake pattern or stress on the animal, resembling the effects of feed restriction (Huzzey et al., 2006; Keys, Pearson & Thompson, 1978). The combination of stocking density and fiber content in the diet could explain differences observed in relative abundance of unclassified members of Succinivibrionaceae; however, the effects of this reduction on the ruminal environment or animal performance are unknown and warrant further investigation.

Members of Caulobacteraceae, which belong to the phylum Proteobacteria, also differed significantly as a result of differences in peNDF. In the present study, cows supplemented with straw to provide greater peNDF had lower relative abundance of Caulobacteraceae than cows not supplemented with additional straw. Members of Caulobacteraceae are typically found in aquatic environments and in the gut of different animals and other organisms (Poindexter, 1964). Greater relative abundances of members of Proteobacteria in the gut are sometimes associated with diseased states (Auffret et al., 2017; Shin, Whon & Bae, 2015). A study conducted by Auffret et al. (2017) analyzed the effect two different diets, one on pathogenicity and antimicrobial resistant genes in the rumen of cattle. That study found that cattle consuming more concentrate had greater abundances of Proteobacteria, suggesting greater potential for pathogenicity in cattle consuming high-grain diets (Auffret et al., 2017). This has been supported by other research in cattle (Khafipour et al., 2016). The addition of straw to increase peNDF in the present study may have resulted in decreased Caulobacteraceae. This may result in decreased microbial-derived stress-response genes, suggesting that the addition of peNDF ameliorates the negative effects of high-grain diets (Auffret et al., 2017; Khafipour et al., 2016).

## Conclusions

The hypothesis of this study was that greater stocking density would result in an altered rumen bacterial community composition, and would be affected by presence or absence of straw, resulting in greater or lesser peNDF. Stocking density with or without the inclusion of straw did not have an apparent effect on bacterial community diversity; however, differences were observed at both the phylum and genus level. Greater stocking has a demonstrated negative impact on dairy cattle behavior, including decreased time spent at the feed bunk, particularly immediately following milking (Krawczel et al., 2012a), as well as lying time (Krawczel et al., 2012a). Changes in feeding behavior as a result of stocking density may have negative impacts on the rumen microbiome, as previous studies have found that decreased feed intake or feed restriction can alter the rumen microbial populations and communities (Firkins et al., 1987; McCabe et al., 2015). Although negative effects of overstocking can be observed on the behavior of dairy cattle within one to 2 weeks (Krawczel et al., 2012a), more time may be needed to observe more dramatic effects of stocking density on the rumen microbiome, as other research suggests that at least 6 weeks is required to observe transition to stable bacterial community composition (Clemmons et al., 2019). Alterations in the rumen microbiome of dairy cattle undergoing overstocking could have negative effects on feed efficiency and milk production (Jami, White & Mizrahi, 2014); thus, additional analyses with animals exposed to treatments for longer durations of time may yield more extensive changes to the rumen microbiome.

## Additional Information and Declarations

### Data Availability

The following information was supplied regarding data availability: Sequence data is available at the NCBI Sequence Read Archive: PRJNA527780.
